# Pre-S Deletion and Complex Mutations of Hepatitis B Virus Related to Young Age Hepatocellular Carcinoma in Qidong, China

**DOI:** 10.1371/journal.pone.0059583

**Published:** 2013-03-28

**Authors:** Lishuai Qu, Xiaoling Kuai, Taotao Liu, Taoyang Chen, Zhengpin Ni, Xizhong Shen

**Affiliations:** 1 Department of Gastroenterology, Affiliated Hospital of Nantong University, Jiangsu Province, China; 2 Department of Gastroenterology, Zhongshan Hospital, Fudan University, Shanghai, China; 3 Department of Hepatology, Qidong Liver Cancer Institute, Qidong, Jiangsu Province, China; Yonsei University College of Medicine, Republic of Korea

## Abstract

**Background/Aim:**

To investigate the roles of biomedical factors, hepatitis B virus (HBV) DNA levels, genotypes, and specific viral mutation patterns on the progression of hepatocellular carcinoma (HCC) patients below 40 years of age in Qidong, China.

**Methods:**

We conducted a case-control study within a cohort of 2387 male HBV carriers who were recruited from August, 1996. The HBV DNA sequence was determined in 49 HCC and 90 chronic hepatitis (CH) patients below 40 years of age. Mutation exchanges during follow-up in 32 cases were compared with 65 controls with paired serum samples. In addition, a consecutive series of samples from 14 HCC cases were employed to compare the sequences before and after the occurrence of HCC.

**Results:**

After adjustment for age, history of cigarette smoking and alcohol consumption, HBeAg positive, HBV DNA levels ≥4.00 log_10_ copies/mL, pre-S deletion, T1762/A1764 double mutations, and T1766 and/or A1768 mutations were associated with risk of young age HCC. Moreover, the presence of an increasing number of HCC-related mutations (pre-S deletion, T1762/A1764, and T1766 and/or A1768 mutations) was associated with an increased risk of young age HCC. Paired samples analysis indicated that the increased HCC risk for at-risk sequence mutations were attributable to the persistence of these mutations, but not a single time point mutation. The longitudinal observation demonstrated a gradual combination of pre-S deletion, T1762/A1764 double mutations, and T1766 and/or A1768 mutations during the development of HCC.

**Conclusion:**

High HBV DNA levels and pre-S deletion were independent risk factors of young age HCC. Combination of pre-S deletion and core promoter mutations increased the risk and persistence of at-risk sequence mutations is critical for HCC development.

## Introduction

Hepatocellular carcinoma (HCC) is the fifth most common cancer and the third most common cause of cancer-related death in the world [1]. Etiologically, majority of HCC develops in chronic hepatitis B virus (HBV) carriers, especially in East Asia and sub-Saharan Africa, where HBV is endemic. Previous studies have shown that chronic HBV infection was associated with the development of HCC in 60% of patients [2]. It is generally accepted that HBV played a major causative role in the development of HCC in humans [3]. Identification of risk factors for HCC and stratification of patient risk are very important to guide future surveillance strategy. The current recommendations most frequently applied for screening patients with HCC are published by the American Association for the Study of Liver Diseases (AASLD) [4]. The recommendations advise HCC screening Asian male HBV patients elder than 40 years and Asian female HBV patients elder than 50 years. Based on this guideline, young patients (under the age of 40 years) could be excluded from cancer screening programs. However, recent studies have reported a significant prevalence and worse prognosis in young HCC patients [5]–[7]. The cost-effectiveness for screening all the HBV carriers below 40 years of age need to be proved. The alternative strategy was to screen the high-risk subjects in this particular age group of HBV carriers.

The pathogenesis of HCC in HBV infection has been extensively investigated, and various viral risk factors have been identified. Recently, high viral load, pre-S deletion, T1653 mutation in enhancer II (EnhII), V1753 mutation, and T1762/A1764 double mutations in basal core promoter (BCP) have been found to be associated with the development of HCC in several reports [8]–[14]. However, to the best of our knowledge, there was no study primarily focused on HBV mutations in young HCC patients. It has been postulated that there may be different mechanisms of hepato-carcinogenesis according to the age distribution of patients [15]. The data are largely lacking in this group of patients.

The township of Qidong is one of the highest endemic regions for chronic HBV infection and HCC in China. This case-control study was conducted within a large cohort of male HBV carriers in Qidong. The goal of the present study was to assess the risk of specific complex mutation patterns with other viral factors in the development of young HCC (under the age of 40 years).

## Methods

### Study Population

The analysis used data and stored samples from a prospective cohort in Qidong, Jiangsu Province, China [16]. From August 1 to September 30, 1996, a total of 18 000 males aged 20 to 65 years, who were living in 17 townships of Qidong, were invited to participate in this HCC screening study. The health examination at study entry included abdominal ultrasonography (US) and serological tests for hepatitis B surface antigen (HBsAg), hepatitis B e antigen (HBeAg), anti-hepatitis C virus (HCV), serum levels of alanine aminotransferase (ALT), and serum alpha-fetoprotein (AFP). A total of 2387 males who were seropositive for HBsAg and free of HCC at recruitment were followed up until October 2006. US, conventional liver function, and AFP levels were tested every 6–12 months. Patients underwent intensive surveillance with computed tomography (CT), magnetic resonance imaging (MRI), and/or hepatic angiography if there was any suspicious abnormality on US or the AFP level was greater than 20 ng/mL. At recruitment, each study participant provided informed written consent and a structured questionnaire on sociodemographic characteristics, habits of alcohol and tobacco consumption. Serum samples collected at interview were stored at −70°C before analysis. This study was approved by the research ethics committee at Zhongshan Hospital, Fudan University, Shanghai, China.

### Cases and Controls

The data of HCC were obtained from medical records and searches of computer files of death certification and cancer registry systems. To ensure complete ascertainment, we also contacted relatives to identify cases. As a prospective study, in order to eliminate the possible influence of undiagnosed HCC at recruitment, we excluded the HCC cases that were diagnosed within the first two years of follow-up. Our analysis was restricted to HCC diagnosed from October 1, 1998, to September 30, 2006. During this follow-up period, we confirmed 199 incident HCC patients. A total of 73 patients diagnosed with HCC within the first two years of follow-up were excluded from the analysis, 19 were younger than 40 years. The diagnosis of HCC was based on the following criteria: a histopathological examination; 1 imaging technique and a serum AFP level ≥400 ng/mL; or a positive lesion detected by at least 2 different imaging techniques (US, CT, MRI, and hepatic angiography). Several cases qualified based on more than 1 criterion. Of the 199 patients with HCC, 57 were younger than 40 years (young HCC). For each case, we randomly selected two chronic hepatitis (CH) patients as controls from the cohort of HBsAg carriers who were alive and had not been diagnosed with HCC throughout the follow-up period. The controls were individually matched to the cases by age (within 2 years). A total of 114 controls were recruited. Subjects were excluded if they had poor sequence data (3 cases and 5 controls) or polymerase chain reaction (PCR) failure resulted from low quantity of DNA (below about 500 copies/ml; the detection limit of our nested PCR assay) (4 cases and 14 controls) or a history of antiviral therapy (1 cases and 5 controls). Consequently, a total of 49 cases and 90 controls were included in the analysis.

### Serology

Serum HBsAg, HBeAg and anti-HCV antibody were tested by commercially available enzyme immunoassay kits (Shanghai Kehua Bio-engineering Co. Ltd., China). Serum ALT level was determined by ultraviolet-lactate dehydrogenase (UVLDH) method (Shanghai Kehua Bio-engineering Co. Ltd). The serum HBV DNA levels were determined using the Fluorescein quantitative polymerase chain reaction (FQ-PCR) detection system (Taqmen; Roche US), according to the manufacturer’s instructions. The lower limit of detection was 500 copies/mL.

### Nested Polymerase Chain Reaction and Direct Sequencing of the EnhII/BCP/PC and pre-S Regions

HBV DNA was extracted from 200 µL serum samples using the commercial Kit (Shanghai Shenyou Biotech Company, China). HBV genes of the EnhII/BCP/PC regions were amplified by nested PCR. First-round PCR primers were 5′-CAGCTTG TTTTGCTCGCAGC-3′ (nt 1286-1305) and 5′-GAGTAACTCCACAGAAGCTCC- 3′ (nt 2083-2063). PCR reaction was carried out in 50 µL containing 5 µL 10 × buffer, 4 µL 2.5 mmol/L deoxynucleoside triphosphates (dNTP), 2 µL 10 µmol/L sense and antisense primers, 1.5 U PlatinumTaq DNA polymerase (Invitrogen, shanghai, China). First- round PCR was performed as follows: 95°C for 2 min; 95°C for 30 sec, 56°C for 30 sec, and 68°C for 3 min for 35 cycles; and finally, 68°C for 10 min. 2 µL of the first-round PCR product was reamplified by the same PCR condition as the first-round reaction. Second-round PCR primers were 5′-GTGCACTTCGCTTCACC TCT-3′ (nt 1579–1598) and 5′-TCCACAGAAGCTCCGAATTC-3′ (nt 1941-1922). For pre-S region sequence analysis, pre-S genes were amplified under the same PCR condition described above, except the primers were used. First-round PCR primers were 5′-AAAATTAATTATCCTGCTAGG-3′ (nt 2627-2647) and 5′-GAGAAGTC CACCACGAGTC-3′ (nt 269-251). Second-round PCR primers were 5′-TTTACAAC TCTGTGGAAGGC-3′ (nt 2747-2766) and 5′-GAGTCTAGACTCTGTGGTATTGT G-3′ (nt 255-232). All necessary precautions to prevent cross-contamination were taken, and negative controls were included in each assay. Amplified products were directly sequenced in both the forward and reverse directions using an ABI 3700 sequencer and commercial kit (Applied Biosystems, Foster City, CA).

### HBV Genotyping

HBV genotypes were determined by comparing the sequence of EnhII/BCP/PC and pre-S regions with a set of standard sequences obtained from GenBank. Phylogenetic tree was constructed by software MEGA version 3.1.

### Statistical Analysis

Data are presented as means ± SD, proportions, or median (range). To compare the values between the two groups, χ^2^ or Fisher exact tests were performed for categorical variables and Mann-Whitney *U* tests were used for continuous variables with skewed distributions, respectively. Binary unconditional logistic regression models were used to estimate the odds ratios (ORs) of HCC associated with HBV-related factors and corresponding 95% confidence intervals (CIs). Potential confounders including age, history of cigarette smoking and alcohol consumption were adjusted. Multivariate analyses with stepwise logistic regression were used to determine the independent factors associated with HCC. All statistical tests were two sided. *P*<0.05 was considered statistically significant. All statistical analyses were performed using SPSS 11.5 for Windows (SPSS Inc., Chicago, IL).

## Results

### Clinical Features and Virologic Characteristics of Young HCC Patients and Controls

The clinical features and virologic characteristics for patients with and patients without HCC were presented in table 1. There were no statistically significant differences in age, the histories of cigarette smoking and alcohol consumption between HCC patients and controls.

**Table 1:** Characteristics of hepatocellular carcinoma (HCC) cases and controls.

	HCC patients	Controls	Adjusted odds ratio*	
Variable	n = 49 (%)	n = 90 (%)	(95% CI)	*P*-value
Age (yr)	33.37±2.96	32.81±4.56		0.443
Cigarette smoking	26 (53.1)	43 (47.8)		0.552
Alcohol consumption	29 (59.2)	53 (58.9)		0.973
ALT >45 U/L	23 (46.9)	32 (35.6)	1.567 (0.769–3.194)	0.216
HBeAg positive	21 (42.9)	22 (24.4)	2.326 (1.092–4.955)	0.029
HBV DNA levels				
(log_10_ copies/mL)				
1 (2.69–3.99)	5 (10.2)	26 (28.9)	1.00 (reference)	
2 (4.00–5.99)	24 (49.0)	43 (47.8)	3.107 (1.035–9.328)	0.043
3 (≥6.00)	20 (40.8)	21 (23.3)	6.040 (1.759–20.743)	0.004
Genotype C	45 (91.8)	82 (91.1)	1.142 (0.319–4.081)	0.839
Pre-S deletion	14 (28.6)	11 (12.2)	2.854 (1.174–6.939)	0.021
T1653	15 (30.6)	17 (18.9)	1.856 (0.823–4.189)	0.136
V1753	15 (30.6)	26 (28.9)	1.157 (0.536–2.499)	0.710
T1762/A1764	35 (71.4)	47 (52.2)	2.295 (1.080–4.878)	0.031
T1766 and/or A1768	12 (24.5)	8 (8.9)	3.167 (1.178–8.520)	0.022
A1896	24 (49.0)	34 (37.8)	1.604 (0.791–3.256)	0.190
A1899	5 (10.2)	6 (6.7)	1.604 (0.458–5.616)	0.460

ALT, alanine aminotransferase.

* Adjusted for age, history of cigarette smoking, history of alcohol consumption.

After adjustment for age, history of cigarette smoking and alcohol consumption, the OR for ALT elevation (>45 U/L) was 1.567 (95% CI, 0.769–3.194); seropositivity for HBeAg, 2.326 (95% CI, 1.092–4.955). Compared with participants having serum HBV DNA levels of less than 4.00 log_10_ copies/mL, the adjusted OR was 3.107 (95% CI, 1.035–9.328) for participants with serum HBV DNA levels of 4.00–5.99 log_10_ copies/mL; 6.040 (95% CI, 1.759–20.743), 6.00 log_10_ copies/mL or greater. A significant biological gradient of HCC risk by HBV DNA level from less than 4.00 log_10_ copies/ml to 6.00 log_10_ copies/mL or greater was observed (Table 1).

Genotype C dominated the HBV types in Qidong. HCC patients and control subjects showed the similar distribution pattern for genotype. When we examined HBV DNA sequences in the pre-S and EnhII/BCP/PC regions, pre-S deletion, T1762/A1764, and T1766 and/or A1768 mutations were significantly associated with HCC, showing adjusted ORs from 2.295 to 3.167 (Table 1). The most frequently occurring mutation was T1762/A1764 double mutations. T1653, V1753, A1896, and A1899 mutations were not associated with a higher risk for developing young HCC. Among 139 patients, 25 were infected with pre-S deletion mutation. Compared to control patients, patients with HCC had significant higher frequencies of pre-S deletions. Among the 14 pre-S deletion mutations in the HCC group, three occurred in pre-S1, nine in the 5′ half of the pre-S2 region and four cases had mutations that removed the pre-S2 initiation codon and adjacent sequences. Two of 14 had two deletions (one in pre-S1 and another in the 5′ half of the pre-S2 region). In contrast, of the HBV deletion mutations in the control groups, three occurred in the pre-S1 region, five in the pre-S2 region and three had mutations that removed the pre-S2 initiation codon and adjacent sequences. Compared to patients without pre-S deletion, patients with pre-S deletion had higher proportions of T1766 and/or A1768 mutations [11/25 (44.0%) vs. 9/114 (7.90%), *P*<0.001].

### Multivariate Analysis on the Risk Factors for Young HCC

Unconditional logistic regression analyses showed that HBeAg positive, high viral load (≥4 log_10_ copies/mL), and three sequence mutations (listed in Table 1) were significantly associated the subsequent risk of young HCC. On further calculation using stepwise logistic regression analysis, the followings were found to be independent risk factors of young HCC: high HBV DNA levels and presence of pre-S deletion (Table 2).

**Table 2:** Multivariate analysis of risk factors for the development of HCC.

Factors	Odds ratio (95% CI)	*P*-value
Serum HBV DNA levels	1.925 (1.322–2.803)	0.001
Presence of pre-S deletion	3.979 (1.459–10.850)	0.007

### Association between Young HCC Risk and the Presence of Specific Mutation Patterns

A statistical analysis of the 3 at-risk mutation combinations (pre-S deletion, T1762/A1764 mutations, and T1766 and/or A1768 mutations) was performed in the analysis of the combined risk for HCC. Our data showed that any 2 or 3 mutation combinations were significantly associated with the development of HCC. Compared to patients with wild-type HBV, patients with a single at-risk mutation (OR 2.834; 95% CI, 1.160–6.920), 2 mutation combinations (OR, 4.024; 95% CI, 1.187–13.646), 3 mutation combinations (OR, 8.938; 95% CI, 1.762–45.338) had a higher risk of young HCC. Table 3 demonstrated a significant biological gradient of HCC risk by number of at-risk mutations.

**Table 3:** Association between HCC and the presence of specific mutation patterns of pre-S deletion, T1762/A1764 double mutations, and T1766 and/or A1768 mutations.

	HCC patients	Controls	Adjusted odds ratio*	
Number of mutation	n = 49 (%)	n = 90 (%)	(95% CI)	*P*-value
No mutation	9 (18.4)	39 (43.3)	0.297 (0.128–0.691)	0.005
1	25 (51.0)	39 (43.3)	2.834 (1.160–6.920)	0.022
2	9 (18.4)	9 (10.0)	4.024 (1.187–13.646)	0.025
3	6 (12.3)	3 (3.3)	8.938 (1.762–45.338)	0.008

* Adjusted for age, history of cigarette smoking, history of alcohol consumption.

### Persistence of Sequence Mutation and Young HCC Risk

To analyze association between persistence of a HBV mutation and HCC risk, serum samples at recruitment were also retrieved for sequence analysis. Of all included 139 cases, 32 HCC patients and 65 controls had adequate serum samples collected at recruitment for sequence analysis. The median time interval between the dates of the recruitment samples and the dates of the follow-up samples was 5.7 years (range, 2 to 7.8 years) for cases and 5.2 years (range, 2.5 to 7.5 years) for controls. For at-risk sequence mutations identified to be associated with HCC, detection of a high-risk mutation at both time points was significantly associated with an increased risk of HCC after adjusting for age, history of cigarette smoking, and history of alcohol consumption, while no association with HCC was observed for detection at a single time point mutation (table 4).

**Table 4:** Adjusted ORs for HCC associated with persistence of sequence mutations of HBV.

	At baseline/	HCC patients	Controls	Adjusted odds ratio*	
Variable	at follow-up	n = 32 (%)	n = 65(%)	(95% CI)	*P*-value
Pre-S deletion	−/−	21 (65.6)	57 (87.7)	1.00 (reference)	
	−/+ or +/−	3 (9.4)	3 (4.6)	2.673 (0.477–14.983)	0.264
	+/+	8 (25.0)	5 (7.7)	4.392 (1.277–15.106)	0.019
T1653	−/−	20 (62.5)	53 (81.5)	1.00 (reference)	
	−/+ or +/−	3 (9.4)	5 (7.7)	1.472 (0.309–7.003)	0.627
	+/+	9 (28.1)	7 (10.8)	3.356 (1.089–10334)	0.035
V1753	−/−	21 (65.6)	46 (70.8)	1.00 (reference)	
	−/+ or +/−	4 (12.5)	7 (10.8)	1.198 (0.293–4.900)	0.802
	+/+	7 (21.9)	12 (18.5)	1.294 (0.435–3.853)	0.643
T1762/A1764	−/−	7 (21.9)	29 (44.6)	1.00 (reference)	
	−/+ or +/−	6 (18.8)	10 (15.4)	2.415 (0.636–90168)	0.195
	+/+	19 (59.4)	26 (40.0)	3.203 (1.129–9.088)	0.029
T1766 and/or A1768	−/−	23 (71.9)	59 (90.8)	1.00 (reference)	
	−/+ or +/−	3 (9.4)	3 (4.6)	2.648 (0.469–14.954)	0.270
	+/+	6 (18.8)	3 (4.6)	5.297 (1.210–23.188)	0.027
A1896	−/−	11 (34.4)	27 (41.5)	1.00 (reference)	
	−/+ or +/−	8 (25.0)	11 (16.9)	1.506 (0.468–4.850)	0.492
	+/+	13 (40.6)	25 (38.5)	1.378 (0.501–3.788)	0.535
A1899	−/−	28 (87.5)	60 (92.3)	1.00 (reference)	
	−/+ or +/−	2 (6.2)	2 (3.1)	2.097 (0.272–16.149)	0.477
	+/+	2 (6.2)	3 (4.6)	1.517 (0.225–10.211)	0.668

A total of 32 cases and 65 controls who had a baseline blood sample and a blood sample collected at follow-up were included in analysis.

“−”: absence; “+”: presence.

* Adjusted for age at recruitment, history of cigarette smoking, history of alcohol consumption.

### Longitudinal Observation of HBV Pre-S Deletion and BCP Mutations in Young HCC Patients

Most previous studies on the relationship between HBV mutation and HCC were conducted with a single blood sample. In this study, we also examined the HBV mutations in serum samples spanning the years before and after HCC diagnosis. Among 49 HCC patients with success sequence data, 14 HCC patients with sequential serum samples were selected for the longitudinal investigation of specific mutations (pre-S deletion, T1762/A1764 double mutations, and T1766 and/or A1768 mutations). Table 5 demonstrated the evolution of pre-S deletion and BCP mutations during the progression of HCC. The lack of some information was due to the negative PCR product. There were 14 patients whose HBV sequence could be determined from the serum samples collected at recruitment. Of these 14 patients, 6 showed a gradual occurrence of pre-S deletion, T1762/A1764 double mutations, and T1766 and/or A1768 mutations. Reverse mutation was not observed in any patient. These results, together with those from our case-control study, suggested that any 2 or 3 mutation combinations could be a potential predictive biomarker for HCC.

**Table 5:** Longitudinal observation of specific mutation patterns of pre-S deletion, T1762/A1764, and T1766 and/or A1768 mutations in 14 HCC patients.

Sample	At baseline	2–4 years before HCC	HCC
#1	○―•―○	○―•―○	○―•―•
#2	○―•―○	•―•―○	•―•―○
#3	○―•―○	○―•―○	○―•―○
#4	○―•―○	○―•―○	○―•―○
#5	○―○―○	○―○―○	○―•―○
#6	○―•―•	○―•―•	•―•―•
#7	○―○―○	○―•―○	○―•―○
#8	•―•―•	•―•―•	•―•―•
#9	○―○―○	○―○―○	○―○―○
#10	•―○―○	Negative PCR product	•―•―○
#11	○―•―○	○―•―○	○―•―○
#12	•―○―○	•―○―○	•―○―○
#13	○―•―•	○―•―•	○―•―•
#14	○―○―○	○―○―○	○―○―○

○―○―○, Pre-S―1762/1764―1766/1768; ○: wild-type; •: pre-S deletion, T1762/A1764 double mutations, or T1766 and/or A1768 mutations.

## Discussion

Chronic HBV infection is the main cause of HCC, especially in the Chinese population. The average age at onset of HBV associated HCC is 50 years of age, approximately 10 years earlier than that of onset of HCV-associated HCC [17], [18]. The target population for HCC screening is usually limited to the elderly and the younger age group is therefore neglected in the screening program. However, the incidence of HCC in patients younger than 40 years, especially in high risk populations, is relatively high [19], [20]. Only a small number of articles have so far been written about the epidemiologic, clinical, and histopathologic features of HCC in patients younger than aged 40 years. The relationship between HBV mutation and the development of HCC in this group was rarely demonstrated. The current study may help to clarify the HCC risk factors in young age HBV carriers.

In this case control study from a cohort of HBV carriers, a significantly positive association between high viral load, HBeAg positive, specific sequence mutation and HCC was observed in young males after adjusting for age, history of cigarette smoking, and history of alcohol consumption. Multivariate analysis demonstrated that high HBV DNA levels and presence of pre-S deletion were independent factors associated with the development of HCC in young age HBV carriers. Recently, the role of HBV DNA levels in predicting the progression to HCC has been reported in many studies from mainland of China, Hong Kong, Taiwan, and Japan [8], [9], [21]. Similar to the REVEAL study [8], we found that HBV DNA levels ≥4.00 log_10_ copies/mL started to have increased risk of HCC and HBV DNA levels ≥6.00 log_10_ copies/mL had a further incremental HCC risk. Consistent with previous studies [13], [22], our study has shown the relationship between the presence of pre-S deletion and risk of HCC. The mutations in the BCP region have been widely studied. The relationship between T1762/A1764 double mutations and risk of HCC has been demonstrated in two large cohort studies [10], [23]. Recently, T1766/A1768 mutations have been reported to be associated with the development of HCC [16], [24]. The magnitude of the ORs of HCC associated with the presence of the BCP mutations is generally 2- to 3-fold after adjusting for age, history of cigarette smoking and alcohol consumption. Most previous studies primarily focused on the relationship between certain specific mutation and HCC, it is unclear whether these factors are confounding or a specific combination of these mutations is associated with the development of HCC. In the current study, we then examined the potential value of each mutation or combined mutations for the prediction of HCC. We found that the presence of an increasing number of HCC-related mutations (pre-S deletion, T1762/A1764, and T1766 and/or A1768 mutations) was associated with an increased risk of young age HCC. We then recruited a series of serum samples spanning the years before and after HCC diagnosis. Similar to previous studies [24], the longitudinal observation demonstrated that a gradual combination of pre-S deletion, T1762/A1764 double mutations, and T1766 and/or A1768 mutations during the progression of HCC. On the basis of these data, we speculated that during the course of chronic HBV infection, complex mutations occurred in a sequential and accumulative manner. The accumulation of HBV complex mutations may have a synergistic role in the development of HCC.

Most previous studies on the relationship of HBV mutation and the risk of HCC were conducted with the use of samples taken after the diagnosis of cancer. In this investigation, we also examined the HBV mutations in serum samples at recruitment and after HCC diagnosis. The association between the presence of the HBV mutations and HCC risk could be evaluated from samples collected at recruitment and the diagnosis of HCC in combination. Base exchanges in nucleotide of HBV during follow-up appeared in about 6 to 25% of the subjects. We also found that detection of HBV mutations in both the baseline and subsequent samples at diagnosis of HCC was associated with substantially higher risk than detection of this mutation at a single time point. Thus, the increased HCC risk for carriers of HBV strains harboring the mutations is most likely a result of persistence of such mutations.

It is biologically reasonable that pre-S deletion and BCP mutations could contribute to the risk of HCC. The HBV envelope is composed of 3 forms of HBV surface antigen: large (coded for by the pre-S1/pre-S2/S gene), middle (the preS2/S gene), and small (the S gene) protein. The pre-S regions play an essential role in the interaction with the immune responses because they contain several epitopes for T or B cells [25], [26]. In persistent HBV infection, immune epitope deletion mutants occur, escape the host immune surveillance, and lose important functional sites. The deletion over the pre-S gene may affect the expression of middle and small surface proteins, resulting in intracellular accumulation of large surface protein and viral particles, formation of ground glass hepatocytes. These deletion mutations accumulate in the endoplasmic reticulum and cause endoplasmic reticulum stress signals. Through endoplasmic reticulum stress signaling pathways, the pre-S mutant large HBV surface antigens can induce oxidative stress and lead to oxidative DNA damage of HBV infected hepatocytes. Presence of the oxidative DNA lesions stimulates DNA repair activity; the induced mutagenesis occurs in the genome [27], [28]. It has been proposed that BCP mutations may diminish the production of HBeAg and increase viral replication, which theoretically results in increased host immune responses against the virus, therefore increasing hepatocyte apoptosis and degeneration, which leads to liver injury [29], [30]. In addition, this mutation in BCP may alter the binding ability of trans-regulating nuclear factors (such as CCAAT/enhancer-binding protein-α, the ubiquitous transcription factor Sp1, and hepatocyte nuclear factor 4) and may also lead to amino acid alterations of X protein, affect the function of the X protein, interfere with cell growth control and DNA repair and may contribute to the process of multiple steps in hepatocarcinogenesis [31], [32].

The strengths of this study include the sequence analysis of a series of serum samples from a community-based cohort study, repeated sequence analysis provides data on the long-term stability of viral sequence and helps clarify the temporal relationship between a sequence mutation and the occurrence of HCC. Furthermore, this association between viral factors and HCC is unlikely to be biased by the effect of antiviral therapy because the proportion of participants in this cohort who received such therapy was very low (<1%) and no participants with a history of such therapy were included in the analysis. There are also several limitations in this study. First, although CH patients in the control group were age matched with those in the HCC group, the possibility of developing malignancy in the future cannot be denied. Second, the generalizability of the results is limited because all the study subjects were males, a larger cohort and a longer follow-up time are needed for a similar study in females.

In conclusion, this current study further supports the view that Chronic HBV carriers below 40 years of age should not be neglected and should be included surveillance programs for HCC, especially those high risk subjects infected with HBV of complex sequence mutations. These HBV mutations may serve as useful biomarkers for predicting the clinical outcomes of young patients with chronic hepatitis B. Modifications of regular HCC screening guidelines could be expected to result in earlier disease detection and improved prognosis in young patients.

## References

[1] Llovet JM, Burroughs A, Bruix J (2003) Hepatocellular carcinoma. Lancet 362: 1907–17. 14667750 10.1016/S0140-6736(03)14964-1

[2] Lu SN, Su WW, Yang SS, Chang TT, Cheng KS, et al (2006) Secular trends and geographic variations of hepatitis B virus and hepatitis C virus-associated hepatocellular carcinoma in Taiwan. Int J Cancer 119: 1946–52. 16708389 10.1002/ijc.22045

[3] Chen CJ, Yang HI, Iloeje UH (2009) Hepatitis B virus DNA levels and outcomes in chronic hepatitis B. Hepatology. 49: S72–84. 10.1002/hep.22884 19399801

[4] Bruix J, Sherman M (2005) Management of hepatocellular carcinoma. Hepatology 42: 1208–36. 16250051 10.1002/hep.20933

[5] Yang HI, Lu SN, Liaw YF, You SL, Sun CA, et al (2002) Hepatitis B e antigen and the risk of hepatocellular carcinoma. N Engl J Med 347: 168–74. 12124405 10.1056/NEJMoa013215

[6] Bosch FX, Ribes J, Diaz M, Cleries R (2004) Primary liver cancer: worldwide incidence and trends. Gastroenterology 127: S5–S16. 15508102 10.1053/j.gastro.2004.09.011

[7] Lam CM, Chan AO, Ho P, Ng IO, Lo CM, at al (2004) Different presentation of hepatitis B-related hepatocellular carcinoma in a cohort of 1863 young and old patients - implications for screening. Aliment Pharmacol Ther 19: 771–7. 15043518 10.1111/j.1365-2036.2004.01912.x

[8] Chen CJ, Yang HI, Su J, Jen CL, You SL, et al (2006) Risk of hepatocellular carcinoma across a biological gradient of serum hepatitis B virus DNA level. Jama 295: 65–73. 16391218 10.1001/jama.295.1.65

[9] Chan HL, Tse CH, Mo F, Koh J, Wong VW, et al (2008) High viral load and hepatitis B virus subgenotype ce are associated with increased risk of hepatocellular carcinoma. J Clin Oncol 26: 177–82. 18182659 10.1200/JCO.2007.13.2043

[10] Yang HI, Yeh SH, Chen PJ, Iloeje UH, Jen CL, et al (2008) Associations between hepatitis B virus genotype and mutants and the risk of hepatocellular carcinoma. J Natl Cancer Inst 100: 1134–43. 18695135 10.1093/jnci/djn243 PMC2518166

[11] Chou YC, Yu MW, Wu CF, Yang SY, Lin CL, et al (2008) Temporal relationship between hepatitis B virus enhancer II/basal core promoter sequence variation and risk of hepatocellular carcinoma. Gut 57: 91–7. 17502344 10.1136/gut.2006.114066

[12] Yuen MF, Tanaka Y, Shinkai N, Poon RT, But DY, et al (2008) Risk for hepatocellular carcinoma with respect to hepatitis B virus genotypes B/C, specific mutations of enhancer II/core promoter/precore regions and HBV DNA levels. Gut 57: 98–102. 17483190 10.1136/gut.2007.119859

[13] Chen CH, Hung CH, Lee CM, Hu TH, Wang JH, et al (2007) Pre-S deletion and complex mutations of hepatitis B virus related to advanced liver disease in HBeAg-negative patients. Gastroenterology 133: 1466–74. 17915220 10.1053/j.gastro.2007.09.002

[14] Ito K, Tanaka Y, Orito E, Sugiyama M, Fujiwara K, et al (2006) T1653 mutation in the box alpha increases the risk of hepatocellular carcinoma in patients with chronic hepatitis B virus genotype C infection. Clin Infect Dis 42: 1–7. 16323084 10.1086/498522

[15] Namieno T, Kawata A, Sato N, Kondo Y, Uchino J (1995) Age-related, different clinicopathologic features of hepatocellular carcinoma patients. Ann Surg 221: 308–14. 7536406 10.1097/00000658-199503000-00014 PMC1234574

[16] Qu LS, Liu TT, Jin F, Guo YM, Chen TY, et al (2010) Combined pre-S deletion and core promoter mutations related to hepatocellular carcinoma: A nested case-control study in China. Hepatol Res 41: 54–63. 20973883 10.1111/j.1872-034X.2010.00732.x

[17] El-Serag HB, Mason AC (1999) Rising incidence of hepatocellular carcinoma in the United States. N Engl J Med 340: 745–50. 10072408 10.1056/NEJM199903113401001

[18] Bosch FX, Ribes J, Borras J (1999) Epidemiology of primary liver cancer. Semin Liver Dis 19: 271–85. 10518307 10.1055/s-2007-1007117

[19] Sezaki H, Kobayashi M, Hosaka T, Someya T, Akuta N, et al (2004) Hepatocellular carcinoma in noncirrhotic young adult patients with chronic hepatitis B viral infection. J Gastroenterol 39: 550–6. 15235872 10.1007/s00535-003-1341-2

[20] Kim JH, Choi MS, Lee H, Kim do Y, Lee JH, et al (2006) Clinical features and prognosis of hepatocellular carcinoma in young patients from a hepatitis B-endemic area. J Gastroenterol Hepatol 21: 588–94. 16638104 10.1111/j.1440-1746.2005.04127.x

[21] Ohata K, Hamasaki K, Toriyama K, Ishikawa H, Nakao K, et al (2004) High viral load is a risk factor for hepatocellular carcinoma in patients with chronic hepatitis B virus infection. J Gastroenterol Hepatol 19: 670–5. 15151623 10.1111/j.1440-1746.2004.03360.x

[22] Gao ZY, Li T, Wang J, Du JM, Li YJ, et al (2007) Mutations in preS genes of genotype C hepatitis B virus in patients with chronic hepatitis B and hepatocellular carcinoma. J Gastroenterol 42: 761–8. 17876546 10.1007/s00535-007-2085-1

[23] Yuen MF, Tanaka Y, Fong DY, Fung J, Wong DK, et al (2009) Independent risk factors and predictive score for the development of hepatocellular carcinoma in chronic hepatitis B. J Hepatol. 50: 80–8. 10.1016/j.jhep.2008.07.023 18977053

[24] Guo X, Jin Y, Qian G, Tu H (2008) Sequential accumulation of the mutations in core promoter of hepatitis B virus is associated with the development of hepatocellular carcinoma in Qidong, China. J Hepatol 49: 718–25. 18801591 10.1016/j.jhep.2008.06.026

[25] Chisari FV, Ferrari C (1995) Hepatitis B virus immunopathogenesis. Annu Rev Immunol 13: 29–60. 7612225 10.1146/annurev.iy.13.040195.000333

[26] Ferrari C, Cavalli A, Penna A, Valli A, Bertoletti A, et al (1992) Fine specificity of the human T-cell response to the hepatitis B virus preS1 antigen. Gastroenterology 103: 255–63. 1377142 10.1016/0016-5085(92)91121-j

[27] Chen BF, Liu CJ, Jow GM, Chen PJ, Kao JH, at al (2006) High prevalence and mapping of pre-S deletion in hepatitis B virus carriers with progressive liver diseases. Gastroenterology 130: 1153–68. 16618410 10.1053/j.gastro.2006.01.011

[28] Fan YF, Lu CC, Chen WC, Yao WJ, Wang HC, et al (2001) Prevalence and significance of hepatitis B virus (HBV) pre-S mutants in serum and liver at different replicative stages of chronic HBV infection. Hepatology 33: 277–86. 11124846 10.1053/jhep.2001.21163

[29] Baumert TF, Rogers SA, Hasegawa K, Liang TJ (1996) Two core promotor mutations identified in a hepatitis B virus strain associated with fulminant hepatitis result in enhanced viral replication. J Clin Invest 98: 2268–76. 8941643 10.1172/JCI119037 PMC507676

[30] Kidd AH, Kidd-Ljunggren K (1996) A revised secondary structure model for the 3′-end of hepatitis B virus pregenomic RNA. Nucleic Acids Res 24: 3295–301. 8811080 10.1093/nar/24.17.3295 PMC146111

[31] Kay A, Zoulim F (2007) Hepatitis B virus genetic variability and evolution. Virus Res 127: 164–76. 17383765 10.1016/j.virusres.2007.02.021

[32] Li J, Buckwold VE, Hon MW, Ou JH (1999) Mechanism of suppression of hepatitis B virus precore RNA transcription by a frequent double mutation. J Virol 73: 1239–44. 9882327 10.1128/jvi.73.2.1239-1244.1999 PMC103946

